# The relationship between polycystic ovary syndrome and insulin resistance from 1983 to 2022: A bibliometric analysis

**DOI:** 10.3389/fpubh.2022.960965

**Published:** 2022-07-28

**Authors:** Tong Chen, Yue Yu, Fan Jia, Peijie Luan, Xinmin Liu

**Affiliations:** ^1^Department of Gynaecology, Guang'anmen Hospital, China Academy of Chinese Medical Sciences, Beijing, China; ^2^China Academy of Chinese Medical Sciences, Beijing, China; ^3^Department of Orthopedics, Linqu County Chinese Medicine Hospital, Shandong, China

**Keywords:** polycystic ovary syndrome, insulin resistance, bibliometric analysis, CiteSpace, VOSviewer

## Abstract

**Background:**

Polycystic ovary syndrome (PCOS) is a common clinical disease often associated with insulin resistance (IR). The interaction between PCOS and IR will promote the progress of PCOS and the risk of related complications, harm women's physical and mental health, and increase the social and economic burden.

**Materials and Methods:**

PCOS IR-related works of literature were retrieved through the Web of Science Core Collection (WoSCC) Database and imported into VOSviewer and CiteSpace, respectively, in plain text format to conduct the literature visualization analysis of authors, countries, institutions, highly cited works of literature, and keywords, aiming to reveal the hot spots and trends of PCOS IR fields.

**Results:**

A total of 7,244 articles were retrieved from 1900 to 2022. Among them, the United States has made the largest contribution. Diamanti-Kandarakis E was the author with the most publications, and the University of Athens was the institution with most publications. Keyword analysis showed that PCOS interacts with IR mainly through sex-hormone binding globulin, luteinizing hormone, insulin-like growth factor, oxidative stress, and other mechanisms. In addition, the complications of PCOS complicated with IR are also the focus of researchers' attention.

**Conclusions:**

Through bibliometric analysis, this paper obtains the research hotspot and trend of PCOS IR fields, which can provide a reference for subsequent research.

## Introduction

Polycystic ovary syndrome (PCOS) is a complex disease with highly heterogeneous clinical manifestations, which affects about 6–10% of women of reproductive age worldwide, making them prone to infertility, adverse pregnancy outcomes, endometrial cancer, and other diseases (1, 2). Studies have shown that about 35–80% of PCOS patients have insulin resistance (IR) (3, 4), which means researchers are paying more and more attention to the role of IR in PCOS. IR can not only aggravate the hormone disorder and ovulation disorder of PCOS but also increase the incidence of type 2 diabetes and cardiovascular disease (5–7), which greatly increases the social and economic burden and endangers women's physical and mental health. Therefore, it is of great significance to clarify the pathological mechanism between PCOS and IR and to prevent and treat it as soon as possible.

Bibliometrics analysis is a comprehensive knowledge system integrating mathematics, statistics, and philology with an emphasis on quantification (8). It was first defined by Pritchard in 1969 (9) and has developed rapidly in recent years, providing great convenience for literature reading. The CiteSpace V developed by Chaomei Chen of Drexel University and VOSviewer developed by Van Eck NJ of Leiden University are commonly used in bibliometrics. Through qualitative and quantitative analysis of existing database literature, the contribution of different countries, authors, and institutions, as well as hotspots and trends in the research field can be explored. By using the bibliometrics method, combined with VOSviewer and CiteSpace software, this study has conducted a visual analysis of the literature in the field of PCOS IR, tracking research hot spots and trends, to provide a reference for researchers.

## Materials and methods

### Data sources and search strategy

The data in this paper are retrieved through the Web of Science Core Collection (WoSCC) Database, and the retrieval strategy is as follows: TS = (“Polycystic Ovary Syndrome” OR “Ovary Syndrome, Polycystic” OR “Syndrome, Polycystic Ovary” OR “Stein-Leventhal Syndrome” OR “Stein Leventhal Syndrome” OR “Syndrome, Stein-Leventhal” OR “Sclerocystic Ovarian Degeneration” OR “Ovarian Degeneration, Sclerocystic” OR “Sclerocystic Ovary Syndrome” OR “Polycystic Ovarian Syndrome” OR “Ovarian Syndrome, Polycystic” OR “Sclerocystic Ovaries” OR “Ovary, Sclerocystic” OR “Sclerocystic Ovary”) AND TS = (“Insulin resistance”). The time was set to 1900–2022, and the language chosen was English. Review and journal articles were included in this study. The retrieved articles were exported to a plain text file.

### Bibliometric software

In this study, VOSviewer and CiteSpace were used for bibliometric analysis of PCOS IR. VOS Viewer's strong graphical display ability can clearly show the cooperative relationship between projects (10). In VOSviewer, the node size was proportional to the co-occurrence times and the color represents the cluster. Compared with VOSviewer, CiteSpace has a stronger keyword outburst ability and highlights the trend and change of research hotspots (8).

### Data analysis

Plain text files were imported into VOSviewer software for visual analysis of authors, institutions, and countries and were also imported into the Citespace V 5.8 software for keyword visualization analysis. The Citespace software was used to set the following parameters: Time slicing (from 1983 to 2022), Node types (keywords), Pruning (Pathfinder, pruning sliced networks, pruning the merged networks), Selection Criteria (the value of *K* in g-index is changed to 5), and other parameter settings follow the initial software settings.

## Results

### Analysis of article numbers and trends

**Figure 1:**
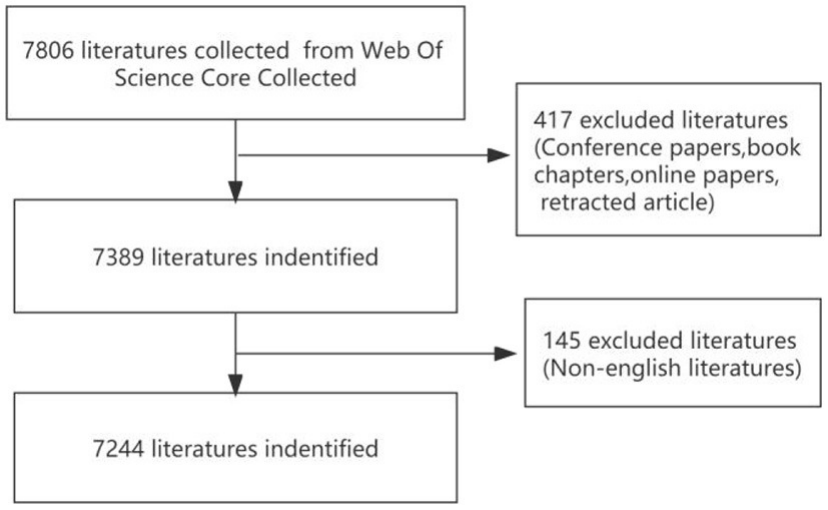
Literature screening flow chart.

**Figure 2:**
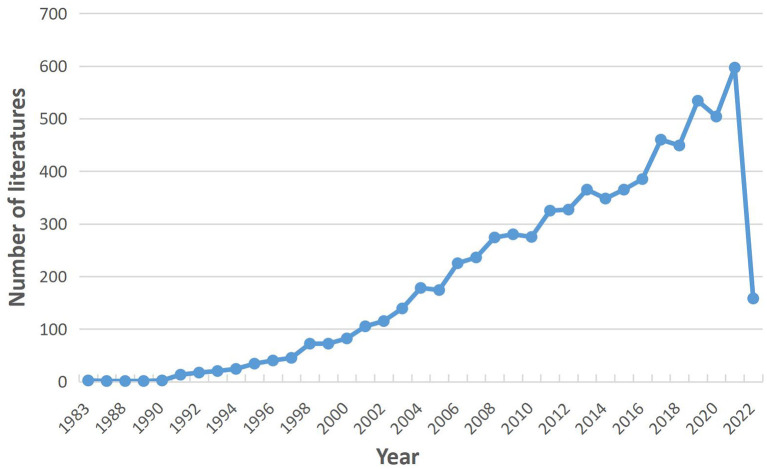
Distribution trend of PCOS IR from 1983 to 2022.

Through screening, a total of 7,244 articles were included in this study (Figure 1). The first article on the PCOS IR field was published in 1983. Since 1991, the number of articles published in the PCOS IR field has continued to increase, reaching a peak of 597 articles in 2021 (Figure 2). As of May 2022, there are 157 articles in the PCOS IR field, and this number will continue to increase.

### Analysis of national cooperation

**Figure 3:**
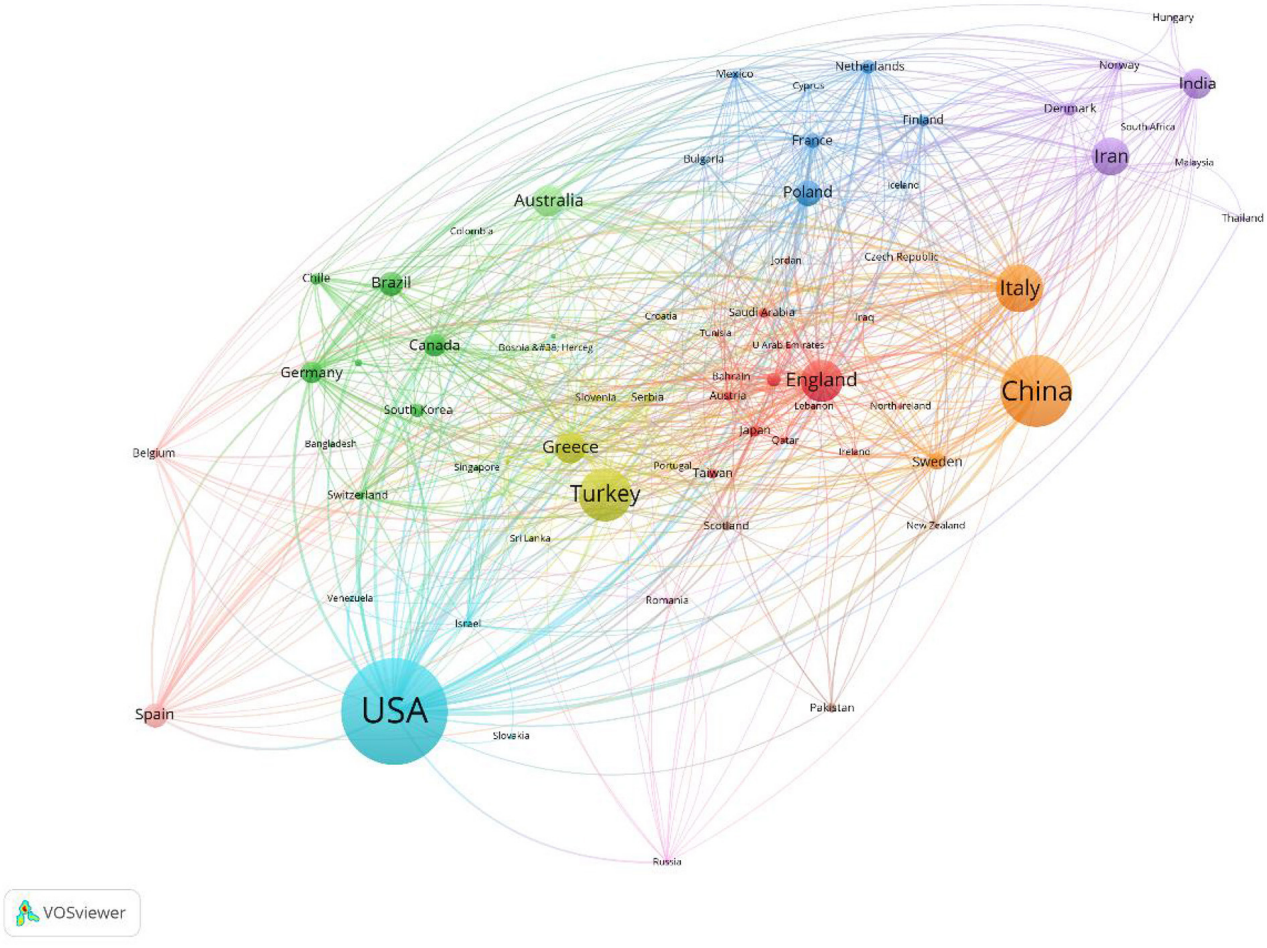
National cooperation visualization map.

Through visual analysis, articles on PCOS IR came from 102 countries (Figure 3). The United States contributed the most, with 1,630 articles (22.5%), followed by China (12.7%) and Turkey (7.1%). The national cooperation visualization map shows that the three countries have very little cooperation, with the United States conducting in-depth cooperation with Poland, France, and other Western countries.

### Analysis of author cooperation

**Figure 4:**
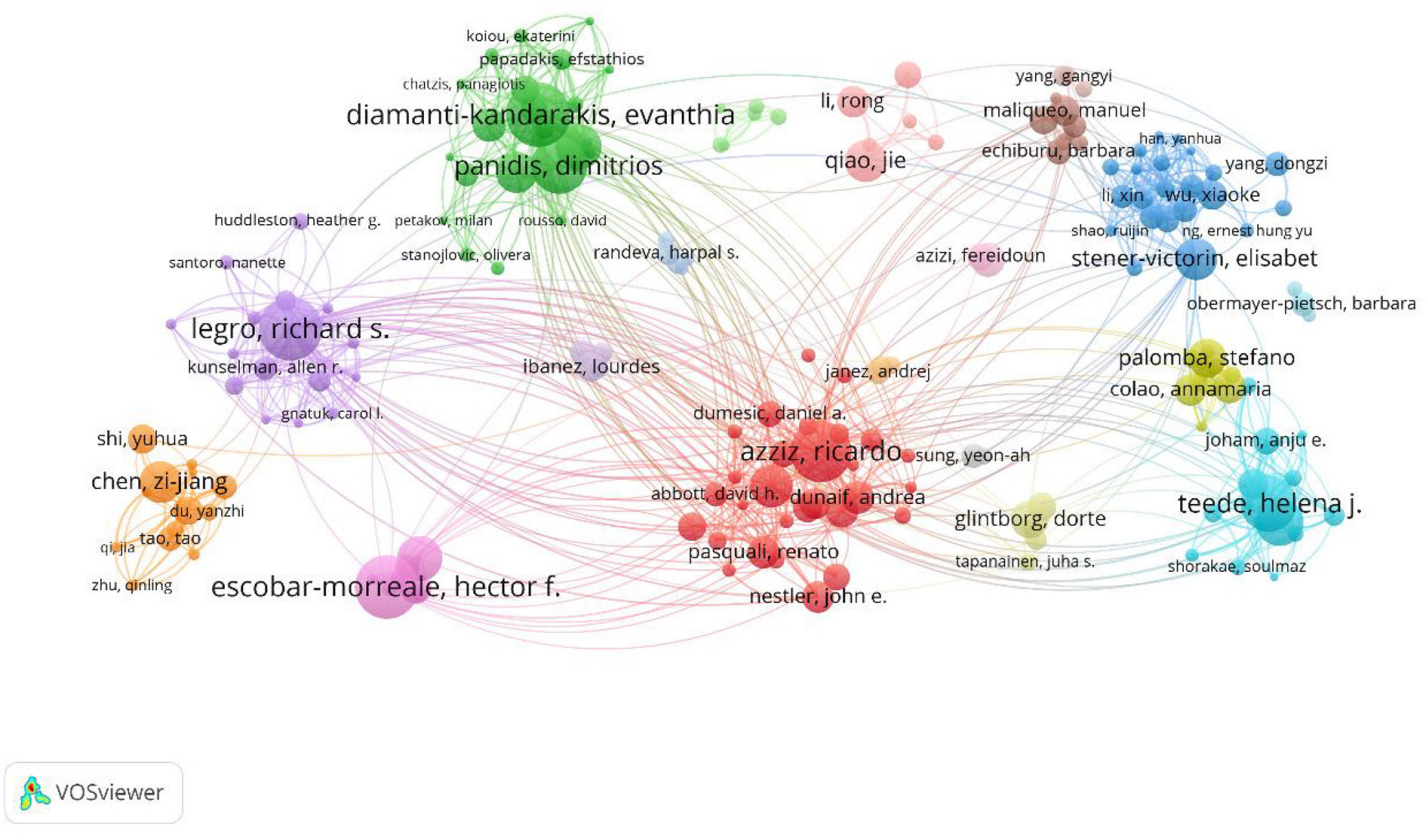
Author cooperation visualization map.

A total of 25,113 authors participated in PCOS IR publications (Figure 4). Diamanti-Kandarakis E ranked first with 64 publications, followed by Escobar-Morreale HF (61) and Legro RS (59). Although Azziz R ranked sixth in the number of articles published, Azziz R's group was at the heart of the network, working closely with other groups.

### Analysis of institutional cooperation

**Figure 5:**
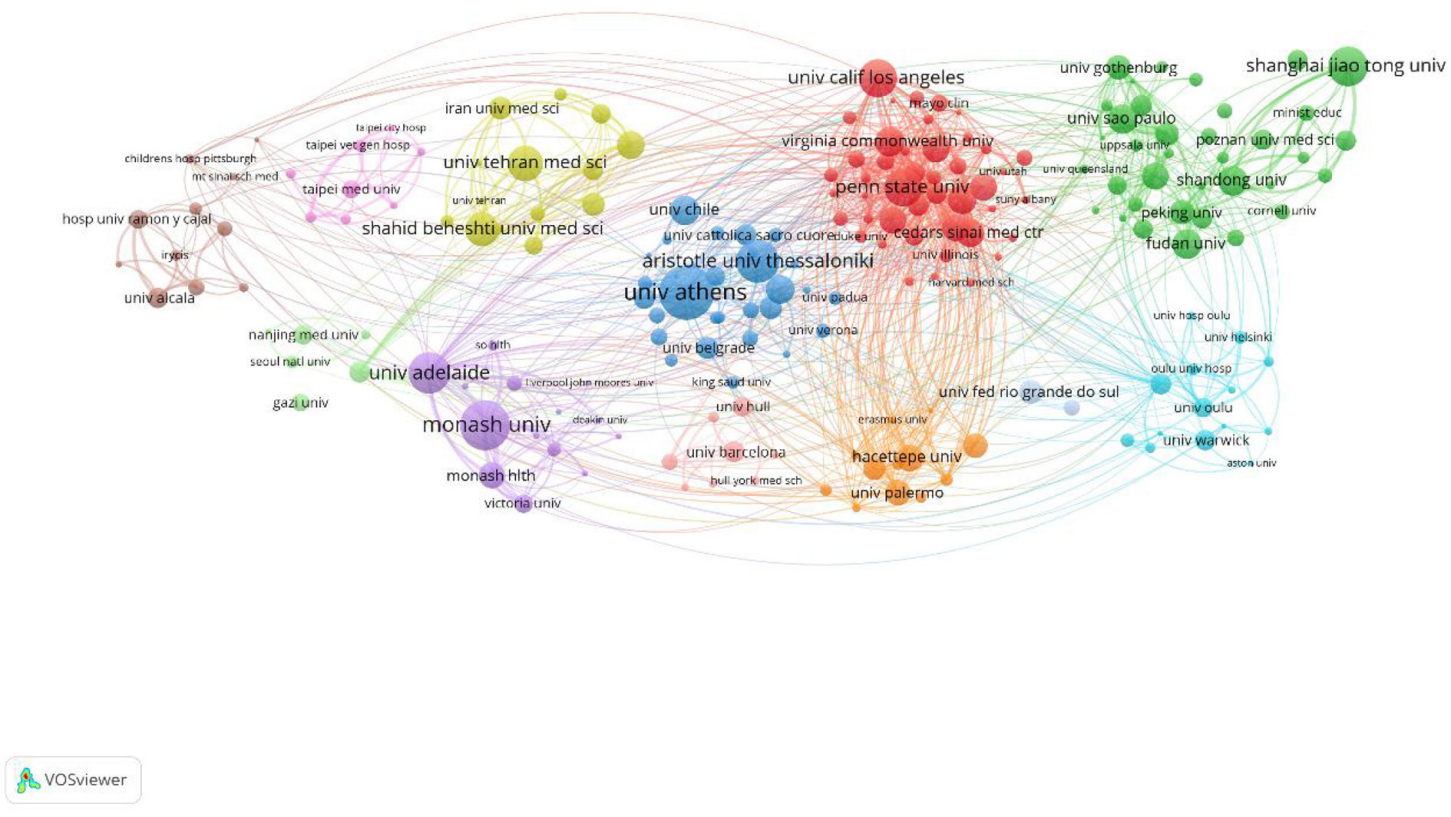
Institution cooperation visualization map.

A total of 4,991 institutions participated in PCOS IR, and the top 10 institutions published 942 articles in total. The University of Athens ranked first with 146 articles, followed by Monash University and Aristotle University Thessaloniki (Figure 5). University of Athens, Aristotle University Thessaloniki, University of Chile, and the University of Belgrade have established close cooperation.

### Top cited publications

The top 10 highly cited literature types include Guideline (6), review (3), and clinical research (1) (Table 1). Due to the heterogeneity of the clinical manifestations of PCOS, the guidelines formulated by various medical associations are not uniform. The high citation of the guidelines represents the demand of researchers for consensus formulation of PCOS.

**Table 1:** Top 20 cited references of PCOS IR fields.

**Ranking**	**Counts**	**Centrality**	**Publication year**	**Author**	**Journal**	**Vol**	**Page**
1	338	0	2004	Rotterdam ESHRE/ASRM-Sponsored PCOS consensus workshop group	Hum. Reprod	19	41
2	306	0	2004	Chang, J	Fertil. Steril	81	19
3	235	0.02	2012	Diamanti-Kandarakis, E	Endocr. Rev	33	981
4	216	0.02	2013	Legro, R.S	J. Clin. Endocr. Metab	98	4565
5	203	0.06	2012	Fauser, B.C.J.M	Fertil. Steril	97	28
6	191	0	2005	Ehrmann, D.A	New. Engl. j. Med	352	1223
7	180	0	2004	Azziz, R	J. Clin. Endocr. Metab	89	2745
8	179	0.02	2010	Wild, R.A	J. Clin. Endocr. Metab	95	2038
9	168	0	2018	Escobar-Morreale, H.F	Nat. Rev. Endocrinol	14	270
10	164	0.02	2009	Azziz, R	Fertil. Steril	91	456
11	158	0	2016	Azziz, R	Nat. Rev. Dis. Primers	2	16057
12	153	0.03	2016	Rosenfield, R.L	Endocr. Rev	37	467
13	153	0.09	2010	March, W.A	Hum. Reprod	25	544
14	151	0.02	2006	Azziz, R	J. Clin. Endocr. Metab	91	4237
15	147	0	1997	Dunaif, A	Endocr. Rev	18	774
16	147	0.02	2013	Stepto, N.K	Hum. Reprod	28	777
17	146	0.01	2016	Bozdag, G	Hum. Reprod	31	2841
18	141	0.01	2005	Apridonidze T	J. Clin. Endocr. Metab	90	1929
19	140	0.01	1999	Legro, R.S	J. Clin. Endocr. Metab	84	165
20	134	0.01	2011	Goodarzi, M.O	Nat. Rev. Endocrinol	7	219

### Analysis of keyword

**Figure 6:**
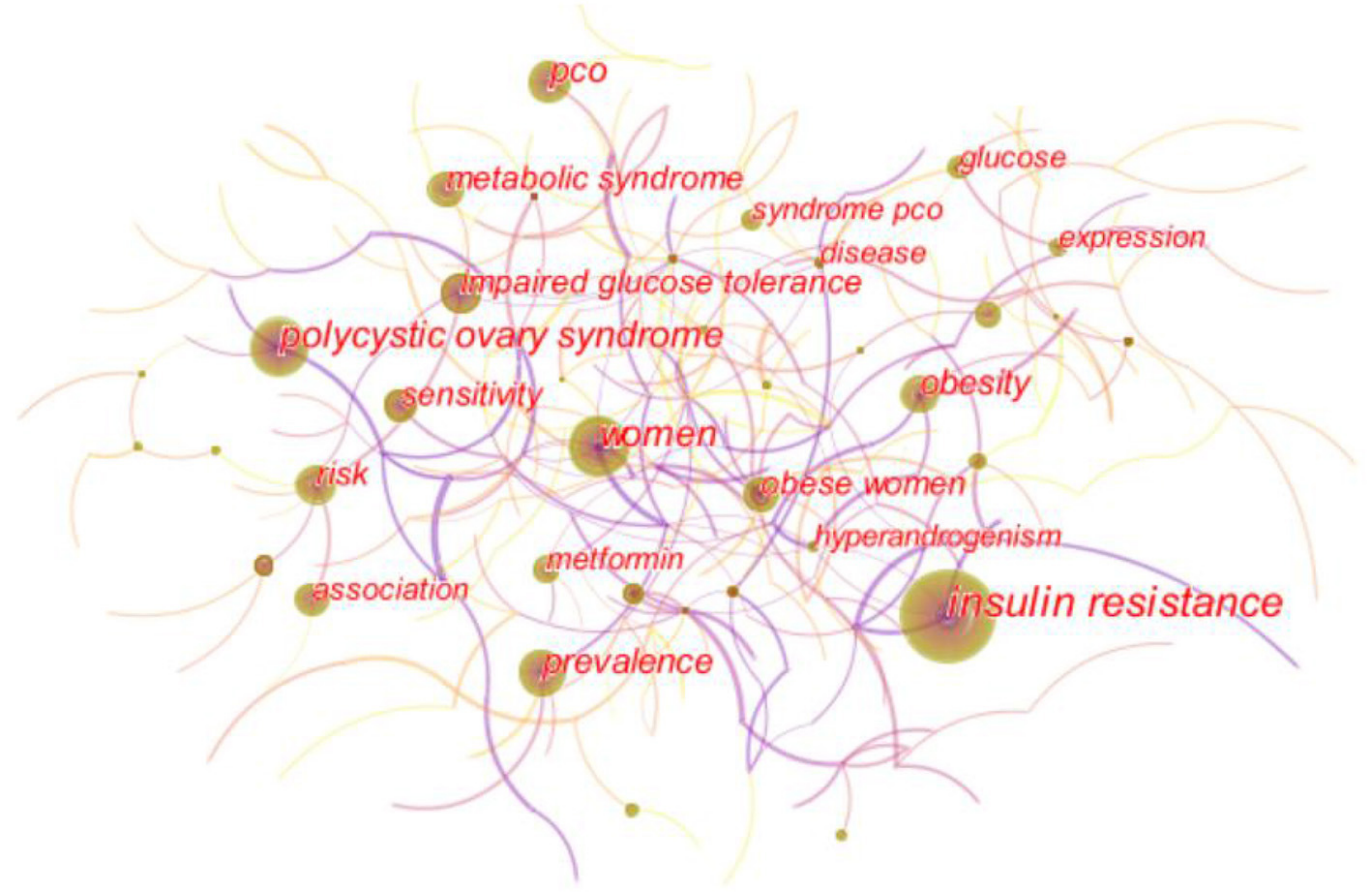
Keyword visualization map.

**Figure 7:**
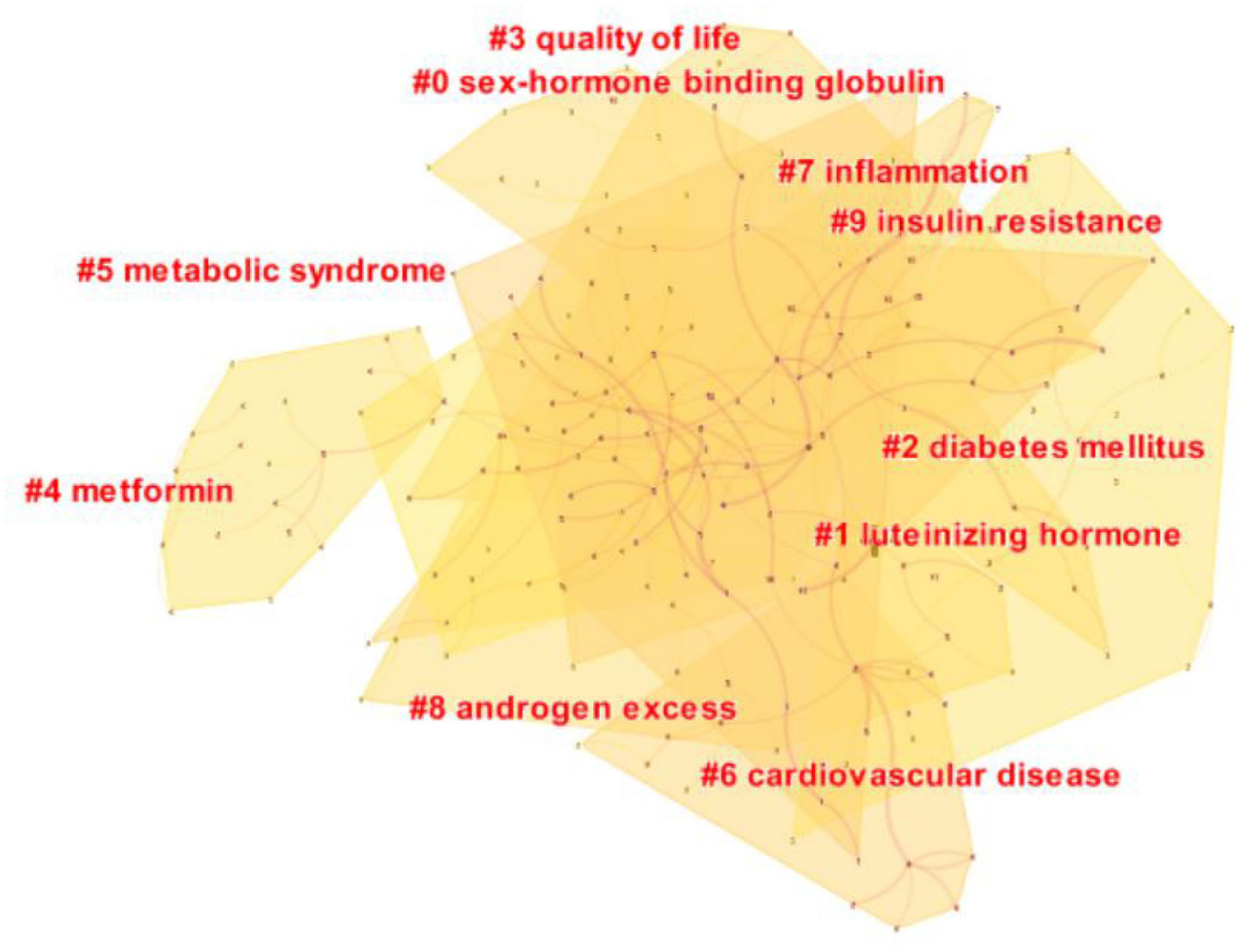
Keywords cluster map.

**Figure 8:**
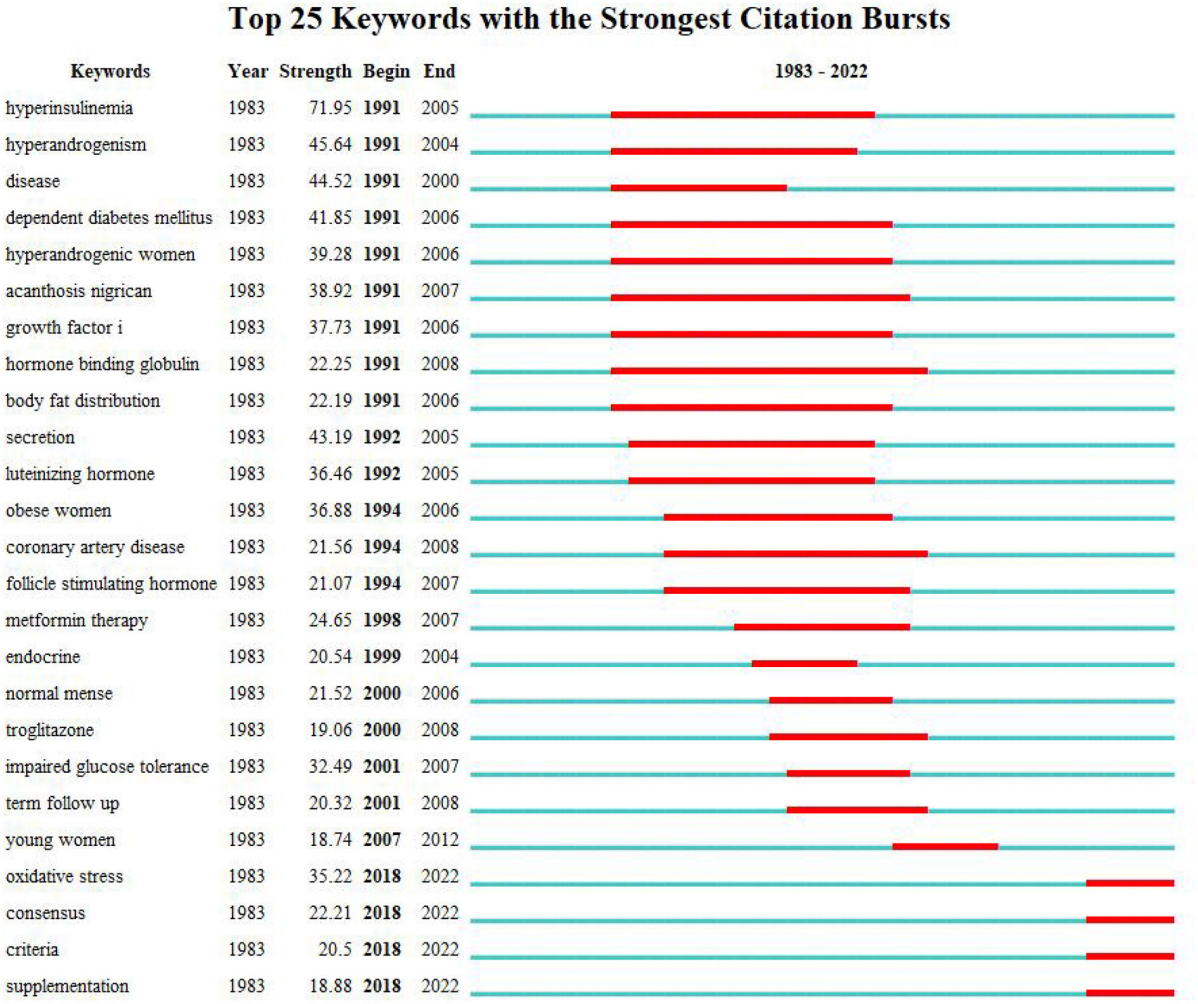
Keywords burst detection map.

As shown in the keyword visualization map (Figure 6), “insulin resistance” was the most frequent keyword, followed by “women,” “polycystic ovary syndrome,” “pco,” “prevalence,” “obesity,” “risk,” “metabolic syndrome,” “impaired glucose tolerance,” and so on. LLR algorithm in Citespace is used to cluster keywords. The top five keywords clusters are “sex-hormone-binding globulin,” “luteinizing hormone,” “diabetes mellitus,” “quality of life,” and “metformin.” (Figure 7). Keywords burst detection map shows that recent focuses of researchers are “oxidative stress,” “consensus,” criteria,” and “supplementation” (Figure 8).

## Discussion

Polycystic ovary syndrome is a reproductive endocrine disease with menstrual disorder, androgen excess, and polycystic ovarian changes as the main clinical manifestations and is the main cause of ovulation dysfunction and infertility. IR and PCOS have a mutually promoting relationship, which not only affects the physical and mental health of PCOS patients but also increases the risk of type 2 diabetes and cardiovascular disease and the social and economic burden. Therefore, it is of great significance to clarify the pathological mechanism between PCOS and IR, and early intervention and treatment for the treatment of PCOS. Bibliometrics analysis can excavate research hotspots in a certain field and display them in the form of network co-occurrence maps, enabling researchers to intuitively and quickly understand the data. In this study, literature bibliometric analysis was conducted on the research field of PCOS IR to explore its development.

The number of PCOS IR papers has shown a steady increase to 2021, which indicates that IR, as an important pathological link in the development of PCOS, continues to receive the attention of researchers. In the first half of 2022, 157 articles were published in the PCOS IR field, which may indicate the number of publications in 2022 will be lower than before. This may be due to incomplete publication or research bottlenecks. While all countries are working together on PCOS IR, the United States made the biggest contribution. This may be related to the high incidence of PCOS in the United States. According to statistics, the incidence of PCOS in the United States is about 5–20%. The annual economic burden caused by PCOS is as high as 3.7 billion dollars (11), and this figure will be much higher if combined with insulin resistance and other long-term complications (12). In addition, it should be noted that the United States, China, Turkey, and other countries with a large number of publications conduct in-depth cooperation to promote the development of this field. Among the authors, Diamanti-Kandarakis E is the author who has published the most, while Azziz R has cooperated the most with other author groups and was in the core position. Surprisingly, educational institutions such as universities are more interested in PCOS IR research than hospitals.

Among the top 10 cited works of literature, there are six works of literature about guidelines that were formulated by Rotterdam European Society of Human Reproduction and Embryology (ESHRE)/American Society for Reproductive Medicine (ASRM)-Sponsored PCOS consensus workshop group, Endocrine Society, Androgen Excess and Polycystic Ovary Syndrome (AE-PCOS) Society. ESHRE/ASRM group thinks two Oligo—and/or anovulation, clinical and/or biochemical signs of hyperandrogenism, and polycystic ovaries to be diagnostic of PCOS, but the role of IR in PCOS has not been emphasized. Endocrine Society endorses ESHRE/ASRM group's diagnostic criteria for PCOS; the AE-PCOS Society has a different opinion, it thinks clinical and/or biochemical signs of hyperandrogenism are the necessary criterion for diagnosis. In addition, both the AE-PCOS Society and the Endocrine Society pay attention to the role of insulin resistance in PCOS and recommend routine screening of glucose tolerance and early treatment. In addition, the only clinical study with top 10 cited references was published by Azziz R in 2004 (13). This clinical study included 400 women aged 18–45 who participated in pre-employment physical examination; the research found that the cumulative prevalence of PCOS was 6.6%, which provided an epidemiological basis for PCOS.

Keyword analysis showed that the pathological mechanism between PCOS and IR was one of the hot spots of researchers' attention. “Sex-hormone-binding globulin,” and “luteinizing hormone” are the top two clusters, representing two common mechanisms of IR increasing PCOS free testosterone level (14, 15): First, insulin receptors in the pituitary gland are triggered to release luteinizing hormone, and second, the synthesis of sex hormone-binding globulin (SHBG) in the liver is inhibited. Increased androgen can also promote the decomposition of adipose tissue, increase the production of free fatty acids and inflammatory factors, and further aggravate IR, causing a vicious cycle (16). According to Figures 6, 8, “Obesity” and “body fat distribution” are also the focus of researchers. Obesity aggravates hyperandrogenemia and insulin resistance, which is the intermediate link between PCOS and IR (17). It is worth noting that thin PCOS is also accompanied by insulin resistance, and the occurrence of IR resistance may be mainly related to visceral fat accumulation (18, 19). According to Figure 8, the heat of “growth factor i” lasted from 1991 to 2006, and the burst intensity was 37.73. The research (20, 21) showed that the serum level of free insulin-like growth factor-1(IGF-1) increases, and insulin-like growth factor-binding protein-1(IGFBP-1) is low in PCOS IR patients; such alternation may drive PCOS follicles to produce excessive sheath androgen. The heat of “oxidative stress” lasted from 2018 to now, and the burst intensity is 35.22. Oxidative stress is a common pathological mechanism of PCOS and IR. Oxidative stress can reduce glucose uptake in musculoskeletal muscle and insulin secretion in pancreatic beta cells to induce IR (22, 23), increase androgen level (24), destroy follicular microenvironment (25), and promote the progress of PCOS and IR. Researchers found that antioxidant therapy can significantly improve the reproductive metabolic disorder of PCOS (26, 27). Metformin has been recommended for these pathological mechanisms, not only because of its ability to increase insulin sensitivity (28), but also because it increases IGF-1 (21, 29), reduces oxidative stress, and partially restores PCOS metabolic and hormonal disorders. Certainly, the pathological mechanism and treatment of PCOS and IR are still worthy of further exploration.

Metabolic disorders and complications secondary to PCOS IR are another hot topic of researchers' attention. “Risk,” “metabolic syndrome,” and “impaired glucose tolerance” in Figure 6, and “diabetes mellitus” and “cardiovascular disease” in Figure 7 are all associated with disease risk. Due to the mutual promotion and crosstalk between IR and androgen overload, patients with PCOS IR have significantly increased risks of diabetes and cardiovascular diseases. It is well known that chronic pancreatic stress under IR can cause impaired glucose tolerance and damage to islet β cells, leading to the occurrence of type 2 diabetes (30, 31). Excessive androgen of PCOS can lead to abnormal vasoconstriction and relaxation function (32, 33), resulting in vascular endothelial dysfunction and aggravating the occurrence of cardiovascular diseases. Guidelines developed by some medical associations began to pay attention to the harm caused by PCOS complications, suggesting screening of glucose tolerance tests, glycated hemoglobin, cardiometabolic risk factors, etc. (34–36). In addition, infertility, irregular menstruation, acne, hirsuteness, obesity, and other manifestations are more common in PCOS IR patients, which makes patients easily complicated with anxiety and depression, resulting in decreased quality of life (37, 38), which should also be given importance by researchers. According to Figure 8, there are also “consensus,” “criteria,” and “supplementation” that have continued since 2018, which reminds researchers of the urgent need for unified standards and consensus development in the field of PCOS IR.

In conclusion, current research focuses on the pathological mechanism between PCOS and IR and the prevention and treatment of long-term risks. The mechanism of PCOS IR is the mutual promotion of hyperandrogenemia and IR, the increase of IGF-1 and the enhancement of oxidative stress, etc. Among them, oxidative stress may be the focus of future research on the mechanism between PCOS and IR. The long-term complications of PCOS IR include diabetes, cardiovascular disease, metabolic syndrome, etc. Early administration of metformin may have positive effects on the treatment of PCOS and the prevention and treatment of its complications, which requires more rigorous clinical trials. It is still the goal of future research to further explore the mechanism between PCOS and IR and to develop therapeutic drugs that can take into account both reproductive and metabolic disorders and psychological abnormalities of PCOS. In addition, it is very necessary to strengthen the cooperative relationship between authors and regions and develop uniform standards and consensus recognized by the industry, which has also been the focus of research in the recent 4 years.

Through bibliometrics, this paper presents the cooperative relationships among authors, countries, and institutions, as well as research hotspots and trends in the field of PCOS IR research, which provides benefits for many researchers. Researchers should optimize their research according to research hotspots, continue to explore the pathological mechanism of PCOS IR, and make continuous efforts to block the disease progression and related complications of patients, maintain the physical and mental health of patients, and reduce the social and economic burden.

However, some limitations of this article have to be considered. First, Since WoS has the most complete citation information, this study only included works of literature in the WoSCC database, which made the literature collection incomplete. Second, there is no unified standard for CiteSpace and VOSviewer software settings, which may cause some deviations in visual analysis.

## Author contributions

TC and YY designed the manuscript. FJ and PL drafted part of the manuscript. XL reviewed the manuscript. All authors approved the final version of the manuscript.

## Funding

This research was funded by the National Natural Science Foundation of China, grant number 81674011, and the Science and Technology Innovation Project of the China Academy of Chinese Medical Sciences, grant number C120221A02404.

## Conflict of interest

The authors declare that the research was conducted in the absence of any commercial or financial relationships that could be construed as a potential conflict of interest.

## Publisher's note

All claims expressed in this article are solely those of the authors and do not necessarily represent those of their affiliated organizations, or those of the publisher, the editors and the reviewers. Any product that may be evaluated in this article, or claim that may be made by its manufacturer, is not guaranteed or endorsed by the publisher.

## References

[1] Mills G Badeghiesh A Suarthana E Baghlaf H. Dahan MH. Associations between polycystic ovary syndrome and adverse obstetric and neonatal outcomes: a population study of 91 million births. Hum Reprod. (2020) 35:1914–21. 10.1093/humrep/deaa144 32644124

[2] Hardiman P Pillay OC Atiomo W. Polycystic ovary syndrome and endometrial carcinoma. Lancet. (2003) 361:1810–2. 10.1016/S0140-6736(03)13409-5 12781553

[3] Amisi CA. Markers of insulin resistance in polycystic ovary syndrome women: an update. World J Diabetes. (2022) 13:129–49. 10.4239/wjd.v13.i3.129 35432749 PMC8984569

[4] Carmina E Lobo RA. Use of fasting blood to assess the prevalence of insulin resistance in women with polycystic ovary syndrome. Fertil Steril. (2004) 82:661–5. 10.1016/j.fertnstert.2004.01.041 15374711

[5] Ehrmann DA Kasza K Azziz R Legro RS Ghazzi MN. Effects of race and family history of type 2 diabetes on metabolic status of women with polycystic ovary syndrome. J Clin Endocrinol Metab. (2005) 90:66–71. 10.1210/jc.2004-0229 15507516

[6] Gomez JMD VanHise K Stachenfeld N Chan JL Merz NB Shufelt C. Subclinical cardiovascular disease and polycystic ovary syndrome. Fertil Steril. (2022) 117:912–23. 10.1016/j.fertnstert.2022.02.028 35512975 PMC10322116

[7] Bogari NM. Genetic construction between polycystic ovarian syndrome and type 2 diabetes. Saudi J Biol Sci. (2020) 27:2539–43. 10.1016/j.sjbs.2020.05.004 32994709 PMC7499096

[8] Shan M Dong Y Chen J Su Q Wan Y. Global Tendency and Frontiers of research on myopia from 1900 to 2020: a bibliometrics analysis. Front Public Health. (2022) 10:846601. 10.3389/fpubh.2022.846601 35359777 PMC8960427

[9] Khalil GM. Gotway Crawford CA. A bibliometric analysis of US-based research on the behavioral risk factor surveillance system. Am J Prev Med. (2015) 48:50–7. 10.1016/j.amepre.2014.08.021 25442231 PMC5285729

[10] van Eck NJ Waltman L. Citation-based clustering of publications using CitNetExplorer and VOSviewer. Scientometrics. (2017) 111:1053–70. 10.1007/s11192-017-2300-7 28490825 PMC5400793

[11] Riestenberg C Jagasia A Markovic D Buyalos RP Azziz R. Health care-related economic burden of polycystic ovary syndrome in the United States: pregnancy-related and long-term health consequences. J Clin Endocrinol Metab. (2022) 107:575–85. 10.1210/clinem/dgab613 34546364

[12] Blonde L. Epidemiology, costs, consequences, and pathophysiology of type 2 diabetes: an American epidemic. Ochsner J. (2001) 3:126–31. 22754388 PMC3385777

[13] Azziz R Woods KS Reyna R Key TJ Knochenhauer ES Yildiz BO. The prevalence and features of the polycystic ovary syndrome in an unselected population. J Clin Endocrinol Metab. (2004) 89:2745–9. 10.1210/jc.2003-032046 15181052

[14] Willis DS Watson H Mason HD Galea R Brincat M Franks S. Premature response to luteinizing hormone of granulosa cells from anovulatory women with polycystic ovary syndrome: relevance to mechanism of anovulation. J Clin Endocrinol Metab. (1998) 83:3984–91. 10.1210/jc.83.11.3984 9814480

[15] Dumesic DA Oberfield SE Stener-Victorin E Marshall JC Laven JS Legro RS. Scientific statement on the diagnostic criteria, epidemiology, pathophysiology, and molecular genetics of polycystic ovary syndrome. Endocr Rev. (2015) 36:487–525. 10.1210/er.2015-1018 26426951 PMC4591526

[16] He FF Li YM. Role of gut microbiota in the development of insulin resistance and the mechanism underlying polycystic ovary syndrome: a review. J Ovarian Res. (2020) 13:73. 10.1186/s13048-020-00670-3 32552864 PMC7301991

[17] Glueck CJ Goldenberg N. Characteristics of obesity in polycystic ovary syndrome: etiology, treatment, and genetics. Metabolism. (2019) 92:108–20. 10.1016/j.metabol.2018.11.002 30445140

[18] Satyaraddi A Cherian KE Kapoor N Kunjummen AT Kamath MS Thomas N. Body composition, metabolic characteristics, and insulin resistance in obese and nonobese women with polycystic ovary syndrome. J Hum Reprod Sci. (2019) 12:78–84. 10.4103/jhrs.JHRS_2_19 31293320 PMC6594114

[19] Romualdi D Versace V Tagliaferri V De Cicco S Immediata V Apa R. The resting metabolic rate in women with polycystic ovary syndrome and its relation to the hormonal milieu, insulin metabolism, and body fat distribution: a cohort study. J Endocrinol Invest. (2019) 42:1089–97. 10.1007/s40618-019-01029-2 30847861

[20] Vasiljević M Prorocić M Dragojević S Tasić L Ganović R Stanimirović B. [The role of insulin-like growth-factor binding proteins in normal and polycystic ovaries]. Srp Arh Celok Lek. (1998) 126:488–94. 9921024

[21] Berker B Emral R Demirel C Corapcioglu D Unlu C Kose K. Increased insulin-like growth factor-I levels in women with polycystic ovary syndrome, and beneficial effects of metformin therapy. Gynecol Endocrinol. (2004) 19:125–33. 10.1080/09513590400007309 15697073

[22] Takeda E Arai H Yamamoto H Okumura H Taketani Y. Control of oxidative stress and metabolic homeostasis by the suppression of postprandial hyperglycemia. J Med Invest. (2005) 52 Suppl: 259–65. 10.2152/jmi.52.259 16366512

[23] Mancini A Bruno C Vergani E d'Abate C Giacchi E Silvestrini A. Oxidative Stress and low-grade inflammation in polycystic ovary syndrome: controversies and new insights. Int J Mol Sci. (2021) 22:10. 10.3390/ijms22041667 33562271 PMC7915804

[24] Yilmaz M Bukan N Ayvaz G Karakoç A Törüner F Cakir N. The effects of rosiglitazone and metformin on oxidative stress and homocysteine levels in lean patients with polycystic ovary syndrome. Hum Reprod. (2005) 20:3333–40. 10.1093/humrep/dei258 16123091

[25] Naigaonkar A Dadachanji R Hinduja I Mukherjee S. Altered redox status may contribute to aberrant folliculogenesis and poor reproductive outcomes in women with polycystic ovary syndrome. J Assist Reprod Genet. (2021) 38:2609–23. 10.1007/s10815-021-02241-x 34041658 PMC8581097

[26] Kazemi M Lalooha F Nooshabadi MR Dashti F Kavianpour M Haghighian HK. Randomized double blind clinical trial evaluating the Ellagic acid effects on insulin resistance, oxidative stress and sex hormones levels in women with polycystic ovarian syndrome. J Ovarian Res. (2021) 14:100. 10.1186/s13048-021-00849-2 34330312 PMC8325180

[27] Pourteymour Fard Tabrizi F Hajizadeh-Sharafabad F Vaezi M Jafari-Vayghan H Alizadeh M Maleki V. Quercetin and polycystic ovary syndrome, current evidence and future directions: a systematic review. J Ovarian Res. (2020) 13:11. 10.1186/s13048-020-0616-z 32005271 PMC6993490

[28] Hernández-Jiménez JL Barrera D Espinoza-Simón E González J Ortíz-Hernández R Escobar L. Polycystic ovarian syndrome: signs and feedback effects of hyperandrogenism and insulin resistance. Gynecol Endocrinol. (2022) 38:2–910. 10.1080/09513590.2021.2003326 34787028

[29] Pawelczyk L Spaczynski RZ Banaszewska B Duleba AJ. Metformin therapy increases insulin-like growth factor binding protein-1 in hyperinsulinemic women with polycystic ovary syndrome. Eur J Obstet Gynecol Reprod Biol. (2004) 113:209–21310. 10.1016/j.ejogrb.2003.09.031 15063962

[30] Diamanti-Kandarakis E Dunaif A. Insulin resistance and the polycystic ovary syndrome revisited: an update on mechanisms and implications. Endocr Rev. (2012) 33:981–1030. 10.1210/er.2011-1034 23065822 PMC5393155

[31] Pani A Gironi I Di Vieste G Mion E Bertuzzi F Pintaudi B. From prediabetes to type 2 diabetes mellitus in women with polycystic ovary syndrome: lifestyle and pharmacological management. Int J Endocrinol. (2020) 2020:6276187. 10.1155/2020/6276187 32587614 PMC7298266

[32] Usselman CW Yarovinsky TO Steele FE Leone CA Taylor HS Bender JR. Androgens drive microvascular endothelial dysfunction in women with polycystic ovary syndrome: role of the endothelin B receptor. J Physiol. (2019) 597:2853–65. 10.1113/JP277756 30847930

[33] Alves JV da Costa RM Pereira CA Fedoce AG Silva CAA Carneiro FS. Supraphysiological levels of testosterone induce vascular dysfunction via activation of the NLRP3 Inflammasome. Front Immunol. (2020) 11:1647. 10.3389/fimmu.2020.01647 32849566 PMC7411079

[34] Wild RA Carmina E Diamanti-Kandarakis E Dokras A Escobar-Morreale HF Futterweit W. Assessment of cardiovascular risk and prevention of cardiovascular disease in women with the polycystic ovary syndrome: a consensus statement by the Androgen Excess and Polycystic Ovary Syndrome (AE-PCOS) Society. J Clin Endocrinol Metab. (2010) 95:2038–49. 10.1210/jc.2009-2724 20375205

[35] Dokras A Saini S Gibson-Helm M Schulkin J Cooney L Teede H. Gaps in knowledge among physicians regarding diagnostic criteria and management of polycystic ovary syndrome. Fertil Steril. (2017) 107:1380–6.e138110. 10.1016/j.fertnstert.2017.04.011 28483503

[36] Dokras A. Heart health in polycystic ovary syndrome: time to act on the data. Fertil Steril. (2022) 117:885–6. 10.1016/j.fertnstert.2022.03.014 35512972

[37] Gibson-Helm M Teede H Dunaif A Dokras A. Delayed diagnosis and a lack of information associated with dissatisfaction in women with polycystic ovary syndrome. J Clin Endocrinol Metab. (2017) 102:604–12. 27906550 10.1210/jc.2016-2963 PMC6283441

[38] Douglas KM Fenton AJ Eggleston K Porter RJ. Rate of polycystic ovary syndrome in mental health disorders: a systematic review. Arch Womens Ment Health. (2022) 25:9–19. 10.1007/s00737-021-01179-4 34499230

